# Determination of the protein content of complex samples by aromatic amino acid analysis, liquid chromatography-UV absorbance, and colorimetry

**DOI:** 10.1007/s00216-022-03910-1

**Published:** 2022-03-23

**Authors:** Kathrin Reinmuth-Selzle, Teodor Tchipilov, Anna T. Backes, Georg Tscheuschner, Kai Tang, Kira Ziegler, Kurt Lucas, Ulrich Pöschl, Janine Fröhlich-Nowoisky, Michael G. Weller

**Affiliations:** 1 Multiphase Chemistry Department, Max Planck Institute for Chemistry, 55128 Mainz, Germany; 2 Division 1.5 Protein Analysis, Federal Institute for Materials Research and Testing (BAM), 12489 Berlin, Germany

**Keywords:** Protein quantification, Aromatic amino acid analysis, LC-UV absorbance, Chemical protein modification, Pollen extract, Air particulate matter, Atmospheric aerosol

## Abstract

Fast and accurate determination of the protein content of a sample is an important and non-trivial task of many biochemical, biomedical, food chemical, pharmaceutical, and environmental research activities. Different methods of total protein determination are used for a wide range of proteins with highly variable properties in complex matrices. These methods usually work reasonably well for proteins under controlled conditions, but the results for non-standard and complex samples are often questionable. Here, we compare new and well-established methods, including traditional amino acid analysis (AAA), aromatic amino acid analysis (AAAA) based on the amino acids phenylalanine and tyrosine, reversed-phase liquid chromatography of intact proteins with UV absorbance measurements at 220 and 280 nm (LC-220, LC-280), and colorimetric assays like Coomassie Blue G-250 dye-binding assay (Bradford) and bicinchoninic acid (BCA) assay. We investigated different samples, including proteins with challenging properties, chemical modifications, mixtures, and complex matrices like air particulate matter and pollen extracts. All methods yielded accurate and precise results for the protein and matrix used for calibration. AAA, AAAA with fluorescence detection, and the LC-220 method yielded robust results even under more challenging conditions (variable analytes and matrices). These methods turned out to be well-suited for reliable determination of the protein content in a wide range of samples, such as air particulate matter and pollen.

**Supplementary Information:**

The online version contains supplementary material available at 10.1007/s00216-022-03910-1.

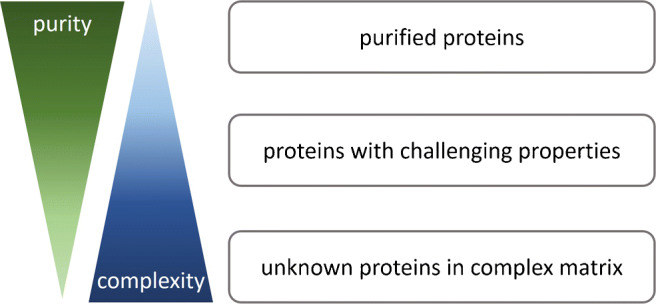

## Introduction

Reliable determination of the protein content in a sample is the fundamental basis for many protein-related studies in biochemical, medical, food chemical, and environmental research like quality control, dose-response studies, enzyme activity determination, nutrition value, and calibration, but the significance and pitfalls of these analytical methods are often highly underestimated.

The methods for quantitative protein determination may differ drastically in relative response factors, matrix dependency, sensitivity, effort for sample preparation, instrumentation, cost, duration, and sample size (picogram to kilogram). For example, the Kjeldahl method is a reference method for protein quantification in food, but it is time consuming and needs large protein amounts (typically 5 g), which makes it less suitable when only small samples are available [1, 2]. In environmental and biomedical research, the most common methods are amino acid analysis, UV absorbance measurements, and colorimetric assays [3, 4] to obtain the total amount of protein contained in a sample. In many cases, the identity of the proteins in complex mixtures is not known and can not be elucidated. Methods for the specific determination of a single or a group of protein(s) or peptide(s), such as mass spectrometry (e.g., [5, 6]) and immunochemical methods [7], are not considered here, because neither the composition nor the respective peptide or protein sequences are usually known in the samples discussed here. Furthermore, the emerging field of metaproteomics [8, 9] is not covered by this article. In this work, more or less pure proteins have been examined mainly for validation purposes.

The gold standard for the accurate determination of the total protein content in complex samples is the total amino acid analysis (AAA). During AAA, the intact proteins are hydrolyzed to individual amino acids usually by acidic hydrolysis. Then, often a precolumn derivatization of the amino acids is performed with, for example, the Edman reagent phenylisothiocyanate (PITC) [10], fluorenylmethyloxycarbonyl chloride (FMOC) [11, 12], o-phthaldialdehyde (OPA) [13], 6-aminoquinolyl-N-hydroxysuccinimidyl carbamate (AQC) [14], 4-fluoro-7-nitro-2,1,3-benzoxadiazole (NBD-F) [15] and others. Subsequently, a liquid-chromatographic step is performed, which should separate the amino acid derivatives of interest. Furthermore, post-column derivatization (e.g., with ninhydrin) is a proven approach [16, 17]. Alternatively, a gas-chromatographic separation can be applied, which has to be combined with one or two derivatization step(s) by, e.g., silanes, chloroformates, and anhydrides [18]. It has to be considered, however, that some amino acids are partially or entirely destroyed during hydrolysis. Hence, a complete amino acid analysis of a sample can hardly be achieved. A correction factor to compensate for these mass deficits may be applied. Recently, a simplified and accelerated version for AAA, termed *Aromatic Amino Acid Analysis* (AAAA), has been presented, based on a reversed-phase separation of the aromatic amino acids phenylalanine (Phe) and tyrosine (Tyr) with UV absorbance or fluorescence detection. This approach does not need any derivatization and can also analyze low protein amounts [19–21]. With this method, the content of Phe or Tyr should be known or a reference sample has to be used for quantification. This reference can be either a representative, complex sample with a known protein content, or a standard protein, such as bovine serum albumin (BSA), which is often used in protein quantification kits. An important advantage of both LC-based AAA approaches is the use of high-performance reversed-phase liquid chromatography (RP-HPLC or UHPLC) as a powerful purification and enrichment step for the predominantly aqueous samples. Other methods for the quantification of total protein, such as the nitric acid method [22], total organic carbon analysis [23], total nitrogen (TN) analysis by combustion [24], sulfur determination by ICP-MS [25], and IR-based quantification [26], have been used less frequently.

Spectroscopic protein measurements are commonly based on the UV absorption of the intact protein at different wavelengths. The peptide bonds as well as the amino acids tryptophan, phenylalanine, tyrosine, and histidine absorb light at around 220 nm, which can be used to determine protein concentrations [27, 28]. Many other substances also absorb light at 220 nm, leaving these methods susceptible to many interferences. In contrast, the absorbance at 280 nm is mainly caused by the aromatic amino acids tryptophan and tyrosine. Thus, the 280-nm absorbance depends directly on the number of aromatic amino acids in the investigated protein and therefore shows a high protein-to-protein variability, but should be less susceptible to interferences than the 220-nm absorbance. However, both UV absorbance measurements at 220 nm or 280 nm can be affected by protein folding, solvent and pH [27].

Other common protein quantification methods are colorimetric assays like the Coomassie Blue G-250 dye-binding assay (Bradford) and the bicinchoninic acid (BCA) assay, which both form colored solutions in response to the investigated protein. In the Bradford assay, the protein-dye interactions are strongly affected by electrostatic interactions of the dye’s sulfonate groups with basic amino acids (arginine and lysine). To a lower extent, histidine and the aromatic amino acids tryptophan, tyrosine, and phenylalanine can stabilize protein-dye interactions [29–31]. In the BCA assay, cupric ions (Cu^2+^) are reduced to cuprous ions (Cu^+^) by the protein via the biuret reaction, and a purple complex between Cu^+^ and bicinchoninic acid is formed. The amino acids cysteine, cystine, methionine, tyrosine, and tryptophan can be easily oxidized and may reduce Cu^2+^ to Cu^+^. In addition, a temperature-dependent reaction of the peptide bond can also produce Cu^+^. Usually, the BCA assay is performed at 37 ^∘^C, but at 60 ^∘^C, the second reaction also contributes to the assay response, which can decrease the protein-to-protein variability [32]. Hence, both colorimetric methods rely on the amino acid composition of the protein in a complex way, and the determined protein concentrations can be easily over- or underestimated if the standard protein is not carefully chosen. Currently, the availability of standard proteins is limited. For most assays, only BSA and, less frequently, gamma-globulins are provided. In most of the available assays, however, certified reference materials (CRM) are not used. Potential interferences of many small molecules or macromolecules for protein assays are sometimes listed in the corresponding manufacturer’s protocols. Thus, determining reliable protein concentration may strongly depend on the amino acid composition as well as on matrix components of a sample.

Protein quantification is affected by the protein composition, chemical modifications, the protein conformation, and the sample matrix [3]. The quantification of chemically modified proteins has rarely been described so far, and the applied methods are often calibrated with non-modified proteins without applying any correction factors. In this work, we have addressed proteins, which had been chemically modified by peroxynitrite (ONOO^−^). The reaction of proteins with ONOO^−^ and other reactive oxygen and nitrogen species (ROS/RNS) leads mainly to the formation of nitrotyrosine and dityrosine (protein dimers and oligomers) [33–36]. Such posttranslational modifications of tyrosine can affect human health and can be used as inflammation and oxidative stress markers [37–45].

In environmental samples such as pollen and air particulate matter, proteins are surrounded by a complex matrix comprising macromolecules and small molecules in concentrations differing by orders of magnitude. For example, the protein content in urban air particulate matter accounts for up to 5% of the sample’s mass, originating from coarse biological particles, like pollen grains, and finer particulates, such as pollen fragments, microorganisms, plant debris and fine soil and dust particles that are mixed and coated with protein [43, 46–48].

In this study, we selected some of the most common protein determination methods such as amino acid analysis, spectroscopic methods based on UV absorbance at 220 nm and 280 nm, and colorimetric methods (BCA and Bradford) to find a representative set of methods, which may be available in most bioanalytical laboratories. Of special note is that we examined two new methods. One is the aromatic amino acid analysis based on phenylalanine and tyrosine, termed AAAA(Phe) or AAAA(Tyr), respectively, using fluorescence detection instead of the former UV absorbance detection [19], which reduces the LOD. A similar method has also been used successfully by Allenspach et al. [20]. The new AAAA(Phe) and AAAA(Tyr) method is much less laborious and costly compared to conventional AAA and provides accurate and robust results. In addition, this method can be performed traceable to certified reference materials, due to the lack of derivatization steps with variable yields. The second new approach was high-performance reversed-phase liquid chromatography (RP-HPLC) with UV absorbance detection at 220 nm and 280 nm, termed LC-220 and LC-280. The separation step can eliminate many interfering substances, which makes this method also suitable for more complex samples such as pollen extracts and atmospheric aerosol samples.

## Materials and methods

### Protein solutions

Bovine serum albumin (> 99.9%, probumin, crystallized, 810014, lot number 121, Merck KGaA, Darmstadt, Germany), chicken avidin (98%, 786-582, VWR International GmbH, Darmstadt, Germany), myoglobin from horse skeletal muscle (M0630-250MG, Sigma-Aldrich Chemie GmbH, Taufkirchen, Germany), jacalin (> 95%, SRP6176-5MG, Sigma-Aldrich Chemie GmbH), bovine apotransferrin (> 95%, PSB-PRO-511, Biozol Diagnostics), and recombinant protein G (> 96%, PSB-PRO-402, Biozol Diagnostics, Munich, Germany) were used. The proteins were dissolved in pure water (Barnstead Gen-Pure xCAD plus water purification system, Thermo Scientific, Braunschweig, Germany) to an expected concentration of 1 mg/mL (gravimetrically), and 500-μL aliquots were stored at − 20 ^∘^C for further analysis. Commercial birch pollen extract (Allergopharma GmbH & Co. KG, Hamburg, Germany) was provided by courtesy of I. Bellinghausen (University Clinics, Mainz, Germany). NIST reference materials (total protein standard, SRM 927e - Bovine Serum Albumin) and amino acids (SRM 2389a - Amino Acids) were used. The characterization of the test proteins is shown in the Electronic Supplementary Material: Protein Characterization.

### Peroxynitrite modification of high-purity BSA

A modified protocol based on Ziegler et al. [38] and Backes et al. [33] was applied. Briefly, 500 μL high-purity BSA solutions (1.0 mg/mL) were prepared with PBS (Thermo Fisher Scientific, Darmstadt, Germany) in a brown reaction tube (Eppendorf, Hamburg, Germany). Then, 12.5- μL ammonium bicarbonate buffer (2 M, 98%, Ph. Eur., BP, Carl Roth, Karlsruhe, Germany) was added and the protein sample was pre-cooled on ice. Different amounts of sodium peroxynitrite (516620-1SET, 200 mM in 4.7% NaOH, Merck KGaA) were added (0.79, 3.95, and 7.9 μL) to yield a molar ratio of peroxynitrite to tyrosine residues of 1/1, 5/1, and 10/1 (ONOO^−^/Tyr). The reaction was performed on ice for 110 min. Sample clean-up was performed with 10 kDa centrifugal filter units (Amicon, Merck Millipore, 0.5 mL). Each sample was washed five times with 500 μL of pure water and centrifuged at 14,000×*g* for 2 min. For sample recovery, the filter was turned upside down, transferred into a clean microcentrifuge tube, and centrifuged at 1000×*g* for 2 min. The final volume of approx. 500 μL was reconditioned with pure water.

### Birch pollen sampling and extraction

Birch pollen was sampled in April 2019 in Mainz, Germany, and stored at room temperature inside a desiccator. Pollen extracts with 10 mg/mL of dry pollen were prepared with pure water. The extraction was performed by shaking with an orbital shaker (100 rpm) at 4 ^∘^C for 14 h. The extracts were filtered through 5-μm and 0.1-μm pore diameter filters (Acrodisc, and PES, Pall GmbH, Dreieich, Germany). In total, 10 × 500-μL aliquots of the aqueous extracts were desalted with Amicon filter devices (Amicon, Ultra 0.5, 3K, Merck Millipore) to a final volume of 500 μL in pure water according to the manufacturers protocol. The desalted extracts were stored at − 20 ^∘^C until further analysis.

### Air particulate matter sampling and extraction

Sampling of air particulate matter was performed on the roof of the Max Planck Institute for Chemistry (Mainz, Germany). Samples of total suspended particles (TSP) and PM_2.5_ were collected on 150-mm glass fiber filters (Type MN 85/90, Macherey-Nagel GmbH, Düren, Germany) using a high-volume sampler (DHA-80, Digitel Elektronik AG, Hegnau, Switzerland) operated at 1000 L/min. The TSP filters were sampled for 7 days in April/May 2018, while the PM_2.5_ filters are 1-day samples (April 2019). For the PM_2.5_ sampling, a 2.5-μm cutting inlet head was installed on the high-volume sampler. To remove any biological contaminations, filters were pre-baked at 330 ^∘^C for 10 h. After sampling, air filter samples were wrapped by pre-baked aluminum bags and stored at − 80 ^∘^C until further analysis.

For the filter extraction, we used a protocol based on Kunert et al. [49] with some modifications. Briefly, each filter was cut into 16 segments. For TSP, three complete air filters (7 days sampling time) were pooled and extracted in different batches. In addition, one baked filter as negative control (filter blank) was extracted. For each extraction batch, 4 out of 16 pieces were extracted with 8 mL of Tris/Gly/SDS (1×) and 32 mL pure water in a sterile 50-mL tube (Falcon tube, Corning, New York, USA). The extracts (480 mL) were vortexed at 2700 rpm for 30 min and filtered through a 5-μm syringe filter. The batch filtrates were pooled and filtered again through a 0.1-μm filter device (Corning PES, New York, USA). For the extraction of PM_2.5_ air filter samples, 4 out of the 16 pieces were kept unextracted for direct hydrolysis and subsequent AAAA(Tyr) and AAA(total) and stored at 4 ^∘^C. Two extraction batches with three filter pieces each were put into a sterile 50-mL tube and 6 mL of Tris/Gly/SDS (1×) buffer and 24 mL of pure water was added and vortexed for 30 min at 2700 rpm. Then, the filters were removed from the tube and the two batches were pooled together (60 mL) and filtered through a 0.1-μm device (Corning PES). Purification and desalting of the pooled TSP and PM_2.5_ air filter extracts were performed with Amicon filter devices (Ultracel, 3K, 15 mL, Merck Millipore LTD). During the TSP air filter clean-up and removing of small molecules (< 3 kDa), the pooled protein extract was concentrated by a factor of 213, while purification of the PM_2.5_ air filter extract yielded a concentration factor of 40. The concentrated and purified aliquots of the air filter extracts were stored at − 20 ^∘^C for further analysis.

### Aromatic amino acid analysis, AAAA(Phe) and AAAA(Tyr)

#### Accelerated hydrolysis

Cysteine (1 mg; ≥ 98%, C8755, Sigma-Aldrich) was dissolved in concentrated hydrobromic acid (HBr, 48 wt.%, 60 μL, ACS reagent, 244260, Honeywell). Sample (10 μL) and cysteine-containing HBr were mixed, sealed, and heated at 150 ^∘^C for 1 h in a suitable hydrolysis vessel. After the hydrolyzed sample has cooled down, it was diluted 1:5 with pure water, centrifuged (20 min, 31,000×*g*), and transferred into HPLC vials. All samples were hydrolyzed in triplicates. This method was developed to shorten the hydrolysis time from 24 h in standard methods to 1 h. The addition of cysteine was performed to avoid the use of inert gas and/or vacuum. A more detailed examination of potential reductants has been published previously [20].

#### Liquid chromatography of underivatized amino acids (AAAA)

Separation of the aromatic amino acids was performed on a reversed-phase column (Agilent Technologies AdvanceBio PeptideMap 2.1 × 150 mm, 2.7 μm) with a precolumn (Agilent AdvanceBio Peptide Map Fast Guard 2.1 mm × 5 mm, 2.7 μm) on the following instrument: Binary pump with integrated degasser (Knauer, P6.1L), autosampler (Knauer, AS3950), column thermostat (Knauer, CT2.1), fluorescence detector (Shimadzu, RF-20Axs). Samples were injected into the instrument in full loop mode (50-μL sample loop, 15-μL flush volume) and underwent chromatographic separation at 0.4 mL/min with aqueous mobile phase A (pure water with trifluoroacetic acid (TFA, 0.2% v/v, HPLC grade, 44630, Alfa-Aesar) and organic mobile phase B (acetonitrile with 0.2% v/v TFA; LC-MS grade, Merck Milli Q-Reference) and a column temperature of 40 ^∘^C. The gradient started at 0 min with 10% B followed by 1.2 min 40% B, 1.3 min 90% B, stayed until 3 min at 90% B and at 3.2 min returned to 10% B and stayed until the end of the run (4.5 min). Fluorescence emission of tyrosine (Tyr) and phenylalanine (Phe) was detected at 272 nm excitation/303 nm emission and 260 nm excitation/280 nm emission wavelengths, respectively. For this approach, the fluorescence detector is configured to perform a wavelength switch at 1.96 min. Calibration was performed externally using a commercial amino acid standard (Supelco AAS18) diluted with pure water. The calibration curves as well as the estimated LODs for AAAA(Phe) and AAAA(Tyr) are shown in Table 1 and Figure S1 (Electronic Supplemetary Material) and an exemplary LC-FLD chromatogram is shown in Figure S2 (Electronic Supplementary Material).

**Table 1:** Limits of detection (LOD) of all applied methods are presented based on BSA

Method	LOD of BSA (mg/L)
AAA	0.02-0.45
AAAA(Phe)	7
AAAA(Tyr)	0.8
LC-220	10
LC-280	20
Bradford, micro	1
BCA	20

For AAA the LODs are estimated from specific LODs of the amino acids. For all calibrations the correlation coefficients were > 99%

#### Protein quantification by AAAA

The protein quantification was based on the assumed phenylalanine and tyrosine composition of the proteins of interest: BSA (27 Phe, 20 Tyr), avidin (28 Phe, 4 Tyr), myoglobin (7 Phe, 2 Tyr), jacalin (12 Phe, 12 Tyr), apotransferrin (29 Phe, 9 Tyr), recombinant protein G (6 Phe, 9 Tyr). To validate the aromatic amino acid analysis method based on phenylalanine used for this study, a sample of a certified bovine serum albumin (NIST standard reference material, SRM 927e, BSA) was examined. The precision and accuracy of the AAAA(Phe) method was evaluated considering two criteria, the relative standard deviation and the recovery of BSA. The certified BSA concentration of the NIST BSA solution based on amino acid analysis is 67.38 ± 1.38 g/L. The measured BSA concentration by our AAAA(Phe) method was 65.2 ± 1.5 g/L based on the measured phenylalanine concentration and the known composition of BSA (27 Phe, 66398.1 g/mol). The determined recovery of 96.8 ± 4.2% based on Phe (260/280 nm) confirmed the method for the use as a reference method. The protein concentration determined by AAAA(Phe) was normalized to 1 mg/mL for all tested protein samples. The LOD was estimated to a protein concentration (BSA equivalent) of 7 mg/L for AAAA(Phe) and 0.8 mg/L for AAAA(Tyr) and is lower compared to the former method based on the UV absorbance of Phe and Tyr [19]. For the birch pollen and air filter extracts, the amino acid composition of the protein mixture is not known. Here, the protein concentration was determined using the amino acid composition of BSA and was calculated as BSA equivalents.

### Standard amino acid analysis (AAA) by AQC derivatization

Total amino acid analysis was performed by Biofidius AG (Bielefeld, Germany). Briefly, from all liquid protein samples (500 μL), three 100-μL aliquots were taken for triplicate measurements. The lyophilized birch pollen extracts were reconstituted with 500 μL of pure water, and three 100-μL aliquots were taken for triplicate measurements. To each aliquot, 100 μL of 12 M HCl was added and incubated at 110 ^∘^C for 24 h. The samples were dried under nitrogen and reconstituted in 100 μL for AQC derivatization and further analysis. The protein quantification was based on the individual amino acid composition of the proteins of interest. Figure S3 (Electronic Supplementary Material) shows the protein concentrations for each individual amino acid. During the hydrolysis cysteine, methionine, tryptophan, and tyrosine are at least partially oxidized or otherwise degraded. Asparagine and glutamine are deamidated and measured together with aspartic and glutamic acid as Asx and Glx, respectively. Thus, the individual compositions of the amino acids alanine, arginine, glycine, histidine, isoleucine, leucine, lysine, phenylalanine, serine, threonine, and proline are used for the determination of the protein content. For the air filter and pollen extracts with largely unknown amino acid composition, the individual amino acids are summed-up and multiplied with a factor of 1.13 to compensate for the missing amino acids. This factor was calculated from the composition of the model protein BSA.

### Standard amino acid analysis (AAA) by OPA/FMOC method for PM_2.5_ air filter samples

#### Standard hydrolysis

This method was adopted from the manufacturer’s protocol (AdvanceBio Amino Acid Analysis, Agilent Technologies). A filter sample (1 out of 16 pieces) was placed in a suitably sized Schlenk tube. Hydrochloric acid (HCl, 6 M, 1.5 mL) was added. The tube was flushed repeatedly with Argon, after which a vacuum (< 10 mbar) was applied. The sample was then heated to 107 ^∘^C for 22 h, after which an aliquot (1 mL) was reduced to dryness (< 10 mbar, 40 ^∘^C, ca. 24 h). Borate buffer (0.4 M) was prepared from sodium tetraborate and adjusted to pH 10.2 using sodium hydroxide solution (10 M). The sample was reconstituted with water (100 μL) and mixed with this borate buffer in a 1:5 ratio, by volume. The diluted sample was centrifuged (15 min, 32,000×*g*) before placing 120 μL (20-μL sample, 100-μL buffer) of it into an HPLC vial.

#### OPA/FMOC derivatization and HPLC

The OPA reagent was prepared by dissolving OPA in borate buffer at a concentration of 10 mg/mL. 3-Mercaptopropionic acid (3-MPA) was added in a threefold molar excess (equivalent to 1.95 μL of 3-MPA per mg of OPA). The FMOC reagent for the derivatization of proline was prepared by dissolving FMOC-Cl in acetonitrile at a concentration of 2.5 mg/mL. The chromatographic separation of the amino acids was performed with the following instrument: Binary pump with integrated degasser (Knauer, P6.1L), autosampler (Knauer, AS3950), column thermostat (Knauer, CT2.1), fluorescence detector (Shimadzu, RF-20Axs). Mobile phase A was prepared as follows: 1.4 g of disodium hydrogen phosphate and 3.8 g of borax decahydrate were dissolved in 1 L of water; pH was adjusted to 8.2 with hydrochloric acid; filtered through a 0.45-μm membrane. Mobile phase B was prepared as follows: ACN, MeOH and water were mixed in 9/9/2 ratio, by volume (225 mL, 225 mL and 50 mL, respectively), and filtered through a 0.45-μm membrane. A RP-AdvanceBio Amino Acid Analysis column (Agilent Technologies, 3.0 × 100 mm, 2.7 μm particle size) with associated guard column was used. Column temperature was set to 40 ^∘^C. The amino acids were derivatized in the autosampler, which was configured to add OPA reagent (10 μL) to the sample vial, mix, wait (10 min), add FMOC reagent (2 μL), mix, wait (3 min) and then inject into the HPLC. Flow was set to 0.63 mL/min and following gradient elution was used: 0 min (2% B), 0.35 min (2% B), 13.4 min (57% B), 13.5 min (100% B), 15.7 min (100% B), 15.8 min (2% B), 25 min (2% B). Fluorescence detection was set to 340 nm/450 nm (excitation/emission) for the first 12 min, then it was changed to 260/325 nm. Experimental data was integrated with Origin’s Peak Analyzer tool using a B-spline baseline. Peaks were filtered by relative intensity and the cut-off was set to 1%. Signal areas were then further evaluated in Microsoft Excel. The individual content of the amino acids alanine, arginine, glycine, histidine, isoleucine, leucine, lysine, phenylalanine, proline, aspartate and asparagine, glutamic acid and glutamine, serine, threonine, tyrosine, valine, and methionine were used for the calculation of the protein content. The individual amino acids are summed-up and multiplied with a factor of 1.07 to compensate for the missing amino acids. This factor was obtained from the composition of the model protein BSA.

### Liquid chromatography (HPLC) with UV detection (LC-220 and LC-280)

Separation of the protein samples was performed with a monomerically bound C18 reversed-phase column (Vydac 238TP, 250 mm × 2.1 mm inner diameter, 5 μm particle size; Grace Vydac, Alltech) on the following instrument (Agilent Technologies): Binary pump with integrated degasser (G1379B), autosampler with thermostat (G1330B, column thermostat (G1316B), photodiode array detector (DAD, G1315C). ChemStation software (Rev. B.03.01, Agilent) was used for system control and data analysis. 10 μL of the samples were injected and underwent chromatographic separation at 0.2 mL/min with aqueous mobile phase A (pure water with trifluoroacetic acid (TFA, 0.1% v/v, HPLC grade, VWR International, Darmstadt, Germany) and organic mobile phase B (acetonitrile, Carl Roth GmbH & Co. KG, Karlsruhe, Germany). The gradient started at 0 min with 3% B followed by a linear gradient to 90% B within 15 min, flushing back to 3% B within 0.2 min, and maintaining 3% B for additional 2.8 min. Column re-equilibration time was 5 min before the next run. Absorbance was monitored at 220 and 280 nm. Each chromatographic run was repeated three times. Calibration of the method was performed with our secondary reference high-purity BSA and is shown in Figure S4 (Electronic Supplementary Material). As an orientation, the LOD was determined with different concentrations of BSA and found to be about 10 mg/L for LC-220 and 20 mg/L for LC-280. However, for each different sample type, a careful examination of the baseline is necessary. LC-220 chromatograms and the purities of the six test proteins are shown in Figure S5 (see Electronic Supplementary Material. As the birch pollen and air filter extracts are a mixture of proteins and other components, the signals between 12.5 and 20 min were integrated and calculated as BSA equivalents.

### Bicinchoninic acid (BCA) assay

A *Pierce BCA Protein Assay Kit* (23225, Thermo Fisher Scientific) was applied according to the manufacturer’s microplate protocol. High-purity BSA was used as standard protein. The BSA solution was diluted to get a calibration curve within the working range of the test tube assay (20–2000 μg/mL). The working reactant (WR) was prepared by mixing 50 parts of bicinchoninic acid (BCA) Reagent A with 1 part of BCA Reagent B. Blank, BSA standard, and protein samples (25 μL) were pipetted in triplicates into a 96-well microplate (Corning, New York, USA), and to each well 200 μL of WR were added and mixed. After a 30 min incubation time at 37 ^∘^C, the absorbance was measured 562 nm on a plate reader (Multiskan GO, Thermo Fisher Scientific).

### Bradford assay

A *Quick Start Bradford Protein Assay* (Bio-Rad Laboratories, Inc.) was performed according to the manufacturer’s standard test tube protocol. Briefly, high-purity BSA was used as standard and diluted to get a calibration curve within the working range of the test tube assay (125–1000 μg/mL). Coomassie Brilliant Blue G-250 dye (1 mL) was mixed with blank, BSA standard and protein samples (20 μL) in a test tube, vortexed and incubated for 30 min at room temperature. The absorbance at 595 nm was measured in triplicates using a spectrophotometer (Multiskan GO, Thermo Fisher Scientific). For the PM_2.5_ air filter extracts the manufacturer’s micro test tube protocol was applied with a working range of 1.25–20 μg/mL and the filter extracts were diluted 1/10 with pure water.

### Quality control by SDS-PAGE with silver stain

The six test proteins (BSA, avidin, myoglobin, recombinant protein G, jacalin, and apotransferrin) were characterized and visualized by silver stained SDS-PAGE (Thermo Fisher Scientific) according to the manufacturer’s protocol. Briefly, protein samples were mixed with an equivalent volume of 2x Laemmli buffer, containing 65.8 mM Tris-HCl (pH 6.8, Carl Roth), 26.3% glycerol (v/v, Carl Roth), 2.1% SDS (Carl Roth), 0.02% bromophenol blue (Sigma-Aldrich) and 5.0% 2-mercaptoethanol (Sigma-Aldrich), and heated at 95 ^∘^C for 5 min. The protein samples (50 ng in 10 μL) and the color-prestained standard marker (10 ng, broad range, 11–245 kDa, New-England Biolabs, Frankfurt, Germany) were loaded onto the gels (Protean Precast, 4–20%, Bio-Rad, Munich, Germany). The manufacturer’s silver stain kit protocol (Thermo Fisher Scientific) was applied. A ChemiDoc system (Bio-Rad) with Image Lab software 5.2.1 (Bio-Rad) was used for image acquisition. The SDS-PAGE gel image can be found in Figure S6 (Electronic Supplementary Material).

### Quality control by MALDI-TOF MS

The sample (1 μL, approx. 1 mg/mL), and the matrix alpha-cyano-4-hydroxycinnamic acid (1 μL, 10 mg/mL in a 1:1 mixture of water and acetonitrile, with 1% trifluoroacetic acid) were spotted onto a 384 spot ground steel target, mixed and left to dry. A Bruker autoflex II smartbeam (Bruker Daltonics GmbH, Leipzig, Germany) with MALDI laser source (355 nm, 200 Hz) was used for all measurements. Measurements were performed in linear, positive mode, up to 1000 scans were accumulated and a mass range up to m/z 200000 was observed. The manufacturer’s software suite (flexControl and flexAnalysis), as well as Origin (OriginLab Corp., MA, USA ) were used for data acquisition and evaluation. The MALDI-TOF MS spectra of the six tested proteins are shown in Figure S7 (see Electronic Supplementary Material).

## Results

Aromatic amino acid analysis based on phenylalanine, termed AAAA(Phe), was used as the reference method, due to the robustness of the analyte and the method. AAAA(Phe) was validated with the NIST reference materials (SRM 927e - Bovine Serum Albumin, Total Protein Standard, and SRM 2389a - Amino Acids) and all methods were normalized to the values obtained by AAAA(Phe) if not stated differently. The concentration of the aqueous high-purity BSA solution used as a calibration standard was determined by AAAA(Phe) and found to be 1.07 ± 0.03 mg/mL. Aliquots of this high-purity BSA solution were used to calibrate all methods.

### High-purity protein as analyte and calibrator

**Fig. 1:**
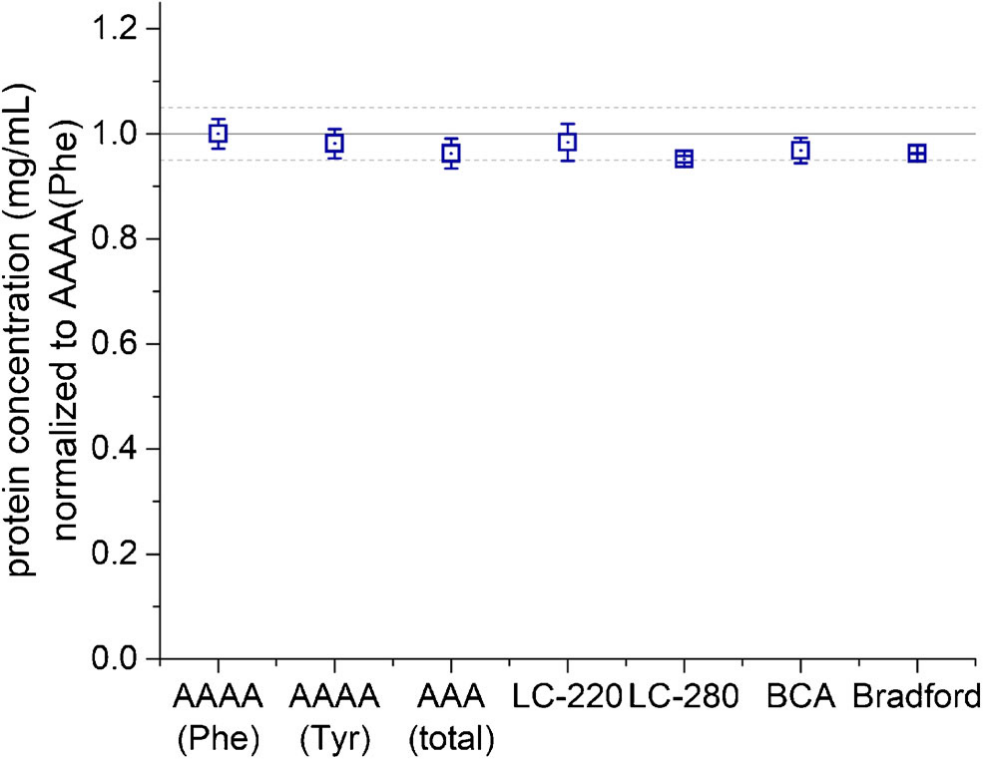
Protein quantification of high-purity bovine serum albumin (BSA) used as sample and calibrator. Protein concentrations were normalized to aromatic amino acid analysis (AAAA) based on phenylalanine (Phe). Arithmetic mean values and standard error of three analytical replicates. Dashed lines indicate a ± 5% interval

High-purity BSA was used as a model protein of interest and calibrator. The protein characterization by SDS-PAGE, MALDI-TOF-MS, C18 liquid chromatography confirmed high-purity BSA as an appropriate calibrator of high purity and quality. All seven methods (AAAA(Phe), AAAA(Tyr), AAA(total), LC-220, LC-280, BCA and Bradford) showed accurate and precise results with a deviation of < 5% (Fig. 1).

### Purified proteins with different properties

**Fig. 2:**
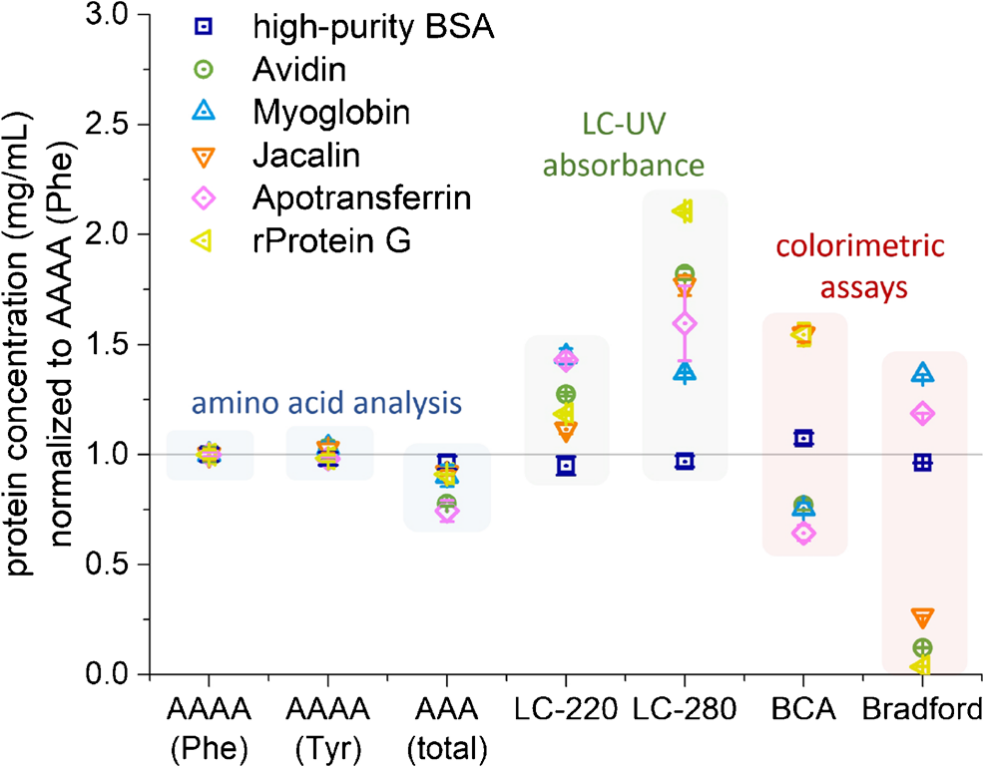
Protein quantification of six proteins with different properties. Protein concentrations were normalized to aromatic amino acid analysis (AAAA) based on phenylalanine (Phe). LC-220, LC-280, BCA, and Bradford were calibrated with high-purity BSA and are BSA equivalent concentrations. Arithmetic mean values and standard error of three analytical replicates are shown. Colored background shadings are to guide the eye

For six relatively pure test proteins, the individual amino acid composition of the protein of interest was used to calculate the protein concentrations based on the obtained amino acid concentrations for the AAA and AAAA methods. Figure 2 shows very similar results with low deviations for all three amino acid analyses. The deviations are slightly higher for the conventional AAA, especially for avidin and apotransferrin. Within the LC-UV absorbance group, high deviations are obtained for LC-280. Here, recombinant Protein G deviated more than 100%, while apotransferrin, jacalin, and avidin showed deviations greater than 50%. LC-220 showed deviations less than 50%. For these methods, BSA equivalents have been calculated. As expected, the BSA value fits perfectly (dark blue squares), whereas proteins with very different extinction coefficients deviate heavily. Within the colorimetric assay group, the BCA assay showed the highest deviation (> 50%) for recombinant protein G and jacalin. The Bradford assay obtained deviations up to 100% for recombinant protein G, avidin and jacalin. Similar to the LC-UV methods, BSA was used for calibration in the colorimetric assays (dark blue squares).

The manufacturers’ protein purities are often specified based on SDS-PAGE. As HPLC usually represents a better method for quantitative measurements, we determined the protein purities based on the main peak percentage of the integrated LC-220 chromatograms. The LC-220 chromatograms of the six tested proteins are shown in Figure S5 (Electronic Supplementary Material). The protein purities were calculated to be 98.7% for BSA, 87.0% for avidin, 97,2% for myoglobin, 82.2% for jacalin, 96.6% for apotransferrin, and 99.1% for recombinant protein G. The LC-220 method and variants thereof might be an alternative (instead of SDS-PAGE) or complementary method for the fast determination of the protein content and purity of protein samples.

### Chemically modified proteins

**Fig. 3:**
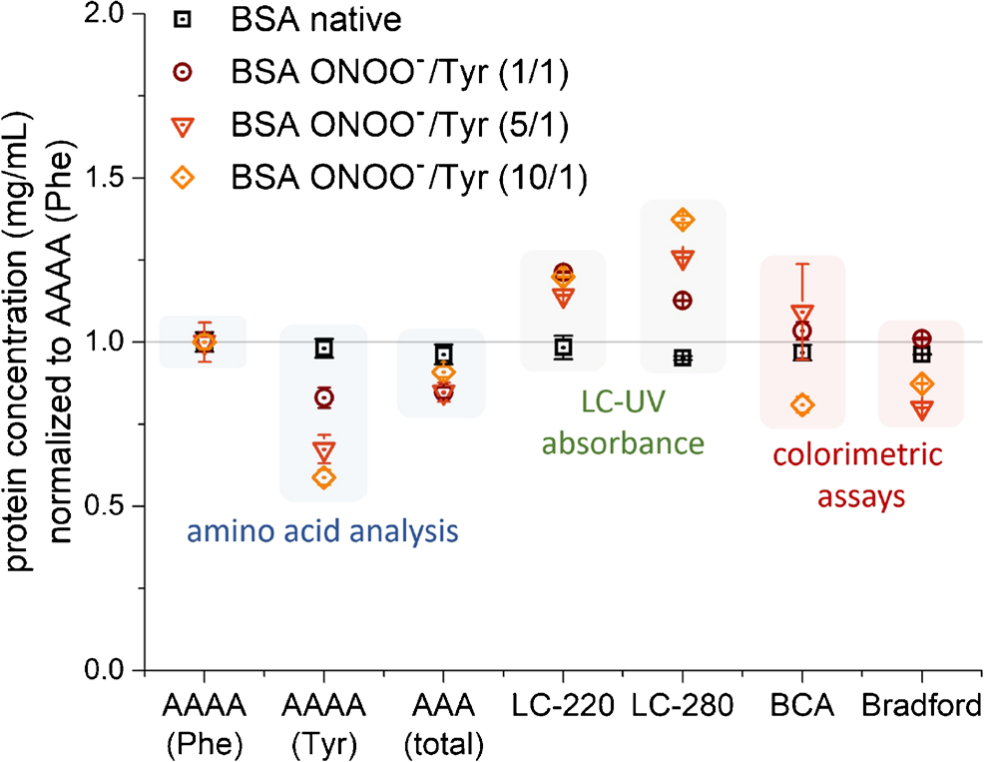
Protein quantification of chemically modified bovine serum albumin. High-purity BSA was treated with increasing molar access of ONOO^−^ over tyrosine (ONOO^−^/Tyr). Protein concentrations were normalized to aromatic amino acid analysis (AAAA) based on phenylalanine (Phe). All methods were calibrated with the secondary reference high-purity BSA. Arithmetic mean values and standard error of three analytical replicates are shown. Colored background shadings are to guide the eye

High-purity BSA was treated with different amounts of peroxynitrite to obtain different molar ratios of peroxynitrite over tyrosine. For chemically modified high-purity BSA, AAAA(Phe) and the AAA (total) results were comparable (Fig. 3). As expected, the AAAA(Tyr) gave lower protein concentrations directly correlated with the molar excess of ONOO^−^ and deviated up to 50% due to the loss of tyrosine. Within the LC-UV absorbance group, the LC-280 method deviated up to 50%, while the LC-220 showed less interference by the chemical modification (< 25%). The BCA and Bradford assays deviated up to 25% and were not correlated with the amount of peroxynitrite.

### Protein mixtures in complex matrices

**Fig. 4:**
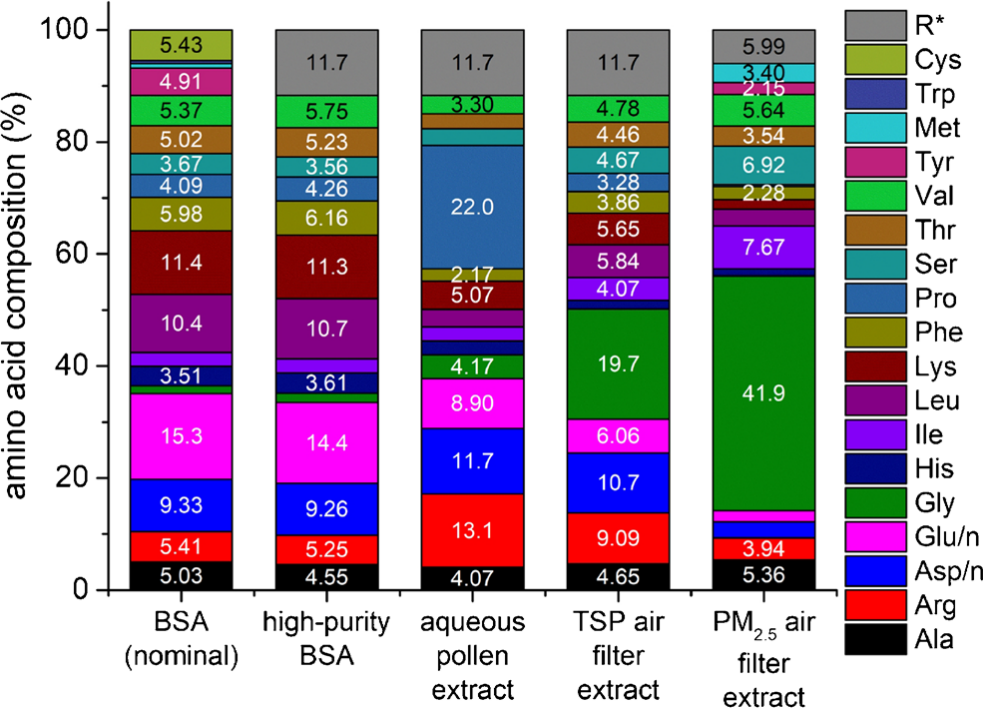
Amino acid composition of high-purity bovine serum albumin (BSA), birch pollen extract, total suspended particles (TSP) and PM_2.5_ air filter extracts as percent by weight. The left column shows the theoretical amino acid composition of BSA. R* represents the remaining amino acids derived from the BSA composition

**Fig. 5:**
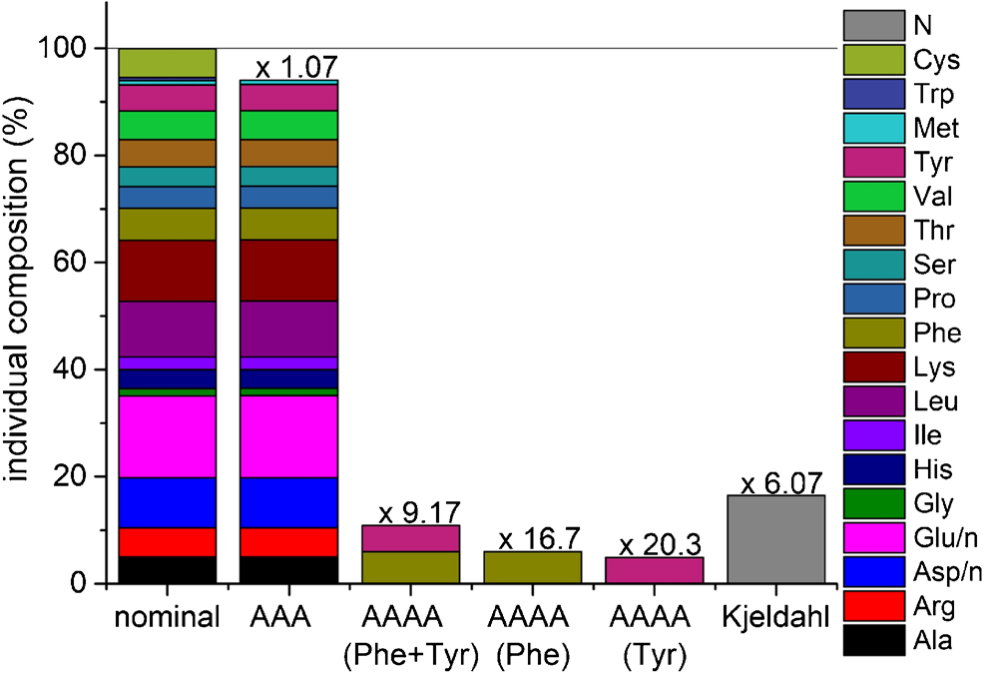
Protein determination with conversion factors exemplified by BSA. Amino-acid-to-protein conversion factors for BSA are shown for different methods. The specific Kjeldahl conversion factor for BSA is 6.07 [50], whereas the default nitrogen-to-protein conversion factor (Jones factor) is 6.25 [51]

In such samples, protein concentration and amino acid composition can vary considerably. This is shown in Fig. 4. The determination of the majority of amino acids seems to be the most accurate method to determine the protein content. A correction factor can be applied to take the lacking amino acids into account. As in other cases, the BSA composition might be used to perform this correction. However, due to cost, time, effort and sample amount limitations, other methods may be more feasible. In these cases, some assumptions about the amino acid composition or other properties have to be made. Usually, the calculation of BSA equivalents should suffice. If a more accurate approach is desired, some representative samples can be examined for their typical AA pattern, which is then transferred to all other samples with the use of an individually calculated conversion factor (Fig. 5). A similar approach is used with the Kjeldahl method [1, 2] in food chemistry, based on the determination of total nitrogen and the application of either a generic conversion factor or a specific one considering the sample type. For the birch pollen and air filter extracts in this work, the protein concentrations obtained by the AAA and AAAA methods were determined based on the amino acid composition of BSA as equivalents. In Fig. 5 some conversion factors are given, which can be used to convert amino acid concentrations into protein concentrations as BSA equivalents. Other pure proteins might also be suitable for calibration. BSA, however, is usually chosen for practical reasons and due to the availability of a certified reference material (CRM). For protein mixtures in complex samples a new approach, LC-220 and LC-280, was tested by integrating the area under the respective chromatograms between 12.5 and 20 min as all test proteins eluted in this retention time window (see Electronic Supplementary Material Figure S4). This time window needs to be verified or adopted for other sample types and chromatographic systems.

**Fig. 6:**
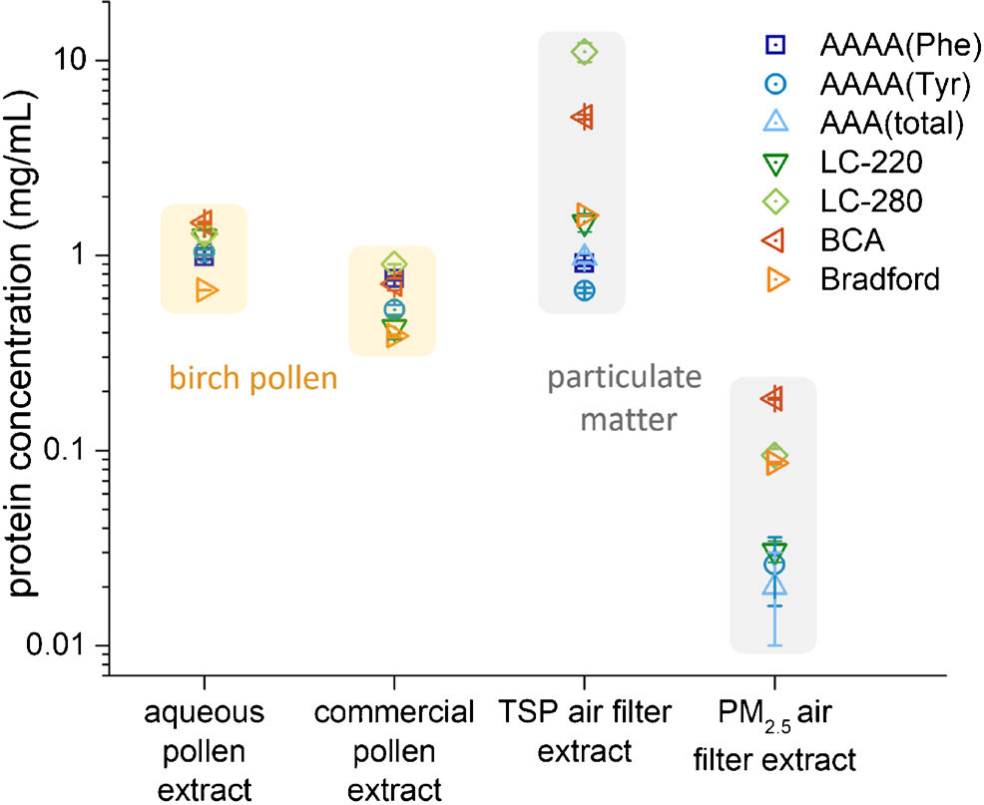
Determination of protein concentrations in real samples. For better comparability of samples with high variability in the protein content, a logarithmic scale was used. In the aqueous pollen extracts following mass fractions are obtained 0.098 (g/g) for AAAA(Phe), 0.104 (g/g) for AAAA(Tyr), and 0.125 (g/g) for LC-220. All methods were calibrated with BSA and are BSA equivalent concentrations. Arithmetic mean values and standard error of three analytical replicates are shown. Background shadings are to guide the eye

In Fig. 6 the protein concentrations in different birch pollen and air filter extracts are shown. For the aqueous birch pollen extract, the obtained protein concentrations are between 0.7 g/L (Bradford) and 1.5 g/L (BCA) with a median of 1.2 g/L. The calculated protein mass fractions (g/g) in pollen were 0.098 for AAAA(Phe), 0.104 for AAAA(Tyr) and 0.125 for LC-220, which means that this dry birch pollen sample contained about 10% of protein. In the commercial birch pollen extract, the protein concentrations were ranging from 0.39 g/L (Bradford) to 0.90 g/L (LC-280). In the TSP air filter extract, the protein results deviated highly, ranging from 0.66 g/L for AAAA(Tyr) to 11.06 g/L (LC-280). The highest values were obtained with LC-280 and all colorimetric assays, which suggests some interferences of matrix components. The PM_2.5_ air filter extract was a particularly challenging real sample, because of the low sample amount, the low protein content and supposedly the very complex composition. For this sample, the sensitivity of AAAA(Phe) was not sufficient to obtain a quantitative value. Compared to the seven-day sampling of the TSP air filter samples, only 24 h sampling was performed with the PM_2.5_ air filter. The protein concentration ranged from 0.02 g/L for AAA to 0.18 g/L for BCA with a median of 0.06 g/L. AAAA(Tyr), AAA(total) and LC-220 were in close agreement, whereas Bradford, BCA and LC-280 deviated heavily.

## Discussion

### High-purity proteins as model analyte and calibrator

Serum albumin is the main protein of human and bovine plasma and is used widely as a standard in analytical assays and molecular biology. We used a solution of highly purified, crystallized bovine serum albumin, termed as high-purity BSA, as our secondary reference. The different methods calibrated with high-purity BSA showed high comparability (± 5%, Fig. 1). Our results show that even simple methods can deliver values of high accuracy when the protein of interest and the protein used for calibration are identical and when matrix effects are negligible. It turned out that, under these controlled conditions, the accuracy and precision of colorimetric assays are not necessarily worse than those of amino acid analysis.

### Purified proteins with different properties

We have chosen some proteins with challenging properties, therefore, these results may represent a worst-case scenario. For these samples, the amino acid analysis group shows results with the lowest deviations. This confirms that AAA still can be considered as the gold standard of quantitative protein analysis. For the LC-UV absorbance group, reasonable results have been obtained for the LC-220 method, except for apotransferrin and myoglobin. Both proteins may contain some iron, which can highly influence UV absorbance. In contrast, the results for LC-280 vary strongly with the highest deviation for recombinant protein G. The individual composition of the aromatic amino acids tryptophan and tyrosine influences the LC-280 signal and seems to be responsible for the high deviations [3]. For UV absorbance-based methods, buffers can affect the results [52], but the use of reversed-phase high-performance liquid chromatography (HPLC) essentially eliminates these interferences, as well as those from other polar molecules. In the colorimetric assay group, the BCA assay shows deviations up to 50%, while the Bradford assay completely failed for three proteins: recombinant protein G, avidin and jacalin. These three proteins contain low proportions of the basic amino acids arginine, lysine, and histidine. The Bradford assay is mainly based on interactions with these basic amino acids, as well as with tryptophan, tyrosine, and phenylalanine to a lesser extent [29–31]. Recombinant protein G does not contain any arginine and histidine residues which can lead to extreme deviations from the expected value. In general, the BCA assay seems to have less protein-to-protein variability compared to the Bradford assay [3]. According to our comparison, the BCA assay is preferable over Bradford, if the use of a colorimetric assay is considered.

### Chemically modified proteins

We treated high-purity BSA with different amounts of peroxynitrite (ONOO^−^) in order to study the effect of chemical protein modifications. Upon ONOO^−^ reaction, tyrosine forms nitrotyrosine and dityrosine, and to a lesser extent hydroxytyrosines [33, 36, 38, 39]. In addition, other amino acids like tryptophan, cysteine, and methionine might also react with the reactive oxygen species ONOO^−^ [39, 40]. As described above, this chemical modification mainly attacks aromatic and redox-sensitive amino acids, which are also essential for many protein determination methods. Reliable and accurate results were obtained for AAAA(Phe), AAA, and LC-220 with a maximal deviation of 25%. As expected, AAAA(Tyr) and LC-280 (Fig. 3) show the strongest deviations directly correlated with the amount of added ONOO^−^. Both methods are strongly dependent on tyrosine. Thus, these methods cannot be recommended for protein quantification of tyrosine-modified or otherwise heavily oxidized proteins. If tyrosine modification levels should be addressed, it is easy to combine AAAA(Phe) with AAAA(Tyr), and LC-220 with LC-280 in one chromatographic run. Then in addition to the protein concentration determination by AAAA(Phe) and LC-220, the tyrosine consumption can be estimated by AAAA(Tyr) and the degree of tyrosine nitration can be obtained by using LC-280 and additional UV absorbance at 357 nm [35]. Although protein modifications are commonly investigated, only a few studies tested how protein modification can affect protein quantification. For example, BCA assay overestimation has been found for lysyl methylation and oxidized beta casein [53, 54], while the glycosylation status of the protein can affect BCA as well as Bradford results [55].

### Protein mixtures in complex matrices

Protein determination of complex samples is the most challenging task due to high uncertainties regarding individual amino acid composition of the proteins, their concentration, and the surrounding matrix.

During the sample preparation of the birch pollen and air filter extracts, particles larger than 0.1 μm and molecules smaller than 3 kDa were removed. Thus, many possible interferences were eliminated, especially the high content of sugars (around 1 mM) in birch pollen [56] or the presence of other redox-active small molecules or metals, which might interfere, e.g., with the BCA assay. In addition, free amino acids can lead to protein overestimation in all AAA methods, which can be prevented (if necessary) by removing small molecules by nanofiltration, dialysis or size exclusion chromatography (SEC). Among the AAA and AAAA methods, the obtained protein concentrations only deviated up to 30%, and the LC-220 method yielded similar results for all tested samples. The highest deviations were found for BCA, Bradford, and LC-280. To examine protein mixtures in complex samples, LC-220 and LC-280 were applied by integrating the respective chromatograms between 12.5 and 20 min as our test proteins fell into this retention time range (see Electronic Supplementary Material Figure S5). This approach seems promising to obtain useful results in a time-saving and simple manner compared to many other conventional methods. For this method it is assumed that most proteins elute in the defined retention time window. Their concentration can be estimated by the UV integral of this group of peaks. It is obvious that this assumption may not hold for all samples and validation would be needed for each application. Orienting analyses might be necessary for different matrices, and for the instrument-specific retention time window.

For the birch pollen extracts, the obtained protein concentrations varied less than expected. Commercial allergen extracts, mainly used in allergy diagnosis, are highly complex due to their natural origin. Here, for the determination of the total protein content in these medicinal products, a range of 50–150% is permitted [57].

Air filter extracts represent a complex mixture of natural and anthropogenic atmospheric aerosols. For example in road dust, metal-containing nanoparticles, soot and amorphous silica nanoparticles have been identified [58]. Previous studies have shown that many aerosol components such as ammonium sulfate, humic-like substances (HULIS) and soot particles can affect protein determination by conventional methods [9, 59]. The metal nanoparticles are known to catalyze redox reactions [60, 61], which can lead to altered BCA responses. For silica nanoparticles, strong interferences with BCA and Bradford assays have been shown. In addition, light scattering and light absorbing properties of nanoparticles can lead to altered UV absorbance results. For example, silica as well as titanium dioxide nanoparticles are shown to absorb light around 300 nm, which might also affect the applied LC-280 [60]. Other possible interfering substances in air filter extracts are HULIS. They are the major component of water-soluble organic carbon and can play important roles in atmospheric processes due to their strong surface activity and light-absorbing capabilities. HULIS are a group of substances with high-molecular weight, low polarity (thus hydrophobic), highly polyconjugated structures and of polyacidic nature [62, 63]. Because of their spectral properties and their redox potential [47, 64], they might also disturb the BCA and the LC-280 method. As the air filter extracts were filtered through a membrane with a pore size of 0.1 μm and desalted with 3 kDa cutoff filter units, we can remove many interfering substances with low molecular weight, such as ammonium sulfate, but nanoparticles (< 0.1 μm) and HULIS may still interfere. The PM_2.5_ air filter extracts represent challenging samples because of the small sample size, the low protein content and the complex composition. Therefore, in this case, AAAA(Phe) did not yield a quantitative result due to the limited protein content in the filter sample used for AAAA(Phe). AAAA(Tyr) has an approximately 10-fold better sensitivity than AAAA(Phe) and might be preferable for low concentrations of protein. Furthermore, potentially more sensitive and selective detection methods, such as mass spectrometry should be considered in the future.

### Evaluation of the applied methods

AAAA(Phe) was used as the reference method in this work. Phenylalanine is relatively robust, largely unaffected by the hydrolysis method or oxidative degradation [3] and seems to be a reliable and good proxy for the total protein concentration. Unlike most other methods based on AAA, AAAA does not require derivatization prior to analysis due to the inherent UV absorbance and fluorescence of these aromatic amino acids. A previous study showed a high accuracy and precision of about 5% for AAAA [19] based on UV detection of Phe and Tyr. The LOD of the further improved aromatic amino acid analysis using fluorimetric detection was estimated to be 7 mg/L of BSA for AAAA(Phe) and 0.8 mg/L of BSA for AAAA(Tyr), which is significantly improved compared to the former UV-based method (16 mg/L of BSA) [19]. According to our study, we can recommend aromatic amino acid analysis (AAAA) as a reliable and accurate method for many proteins of interest. Considering the time- and cost-effectiveness, AAAA is preferable to conventional AAA, which, however, is still an excellent method, if available and affordable.

UV absorbance measurements of protein samples (e.g., 220 and 280 nm) are usually performed by measuring the absorbance with a spectrophotometer. The protein sample is transferred into a cuvette or a microwell plate without a further purification step. However, despite of its popularity, this method, which is also used for RNA and DNA, is highly susceptible to interferences and often leads to misleading results [65]. Provided that a liquid chromatography (FPLC, HPLC, UHPLC) system with UV detection is available, one could benefit from an additional purification step by using reversed-phase chromatography of the aqueous samples. We tested this novel approach to combine UV absorbance with RP-liquid chromatography (LC-220, LC-280). LC-220 shows slightly more deviations (particularly overestimations) than AAAA, but in our cases it led to useful results for the estimation of the total protein for samples of similar composition. The LOD of LC-220 and LC-280 was estimated to be 10 mg/L and 20 mg/L respectively. Surprisingly, LC-280 seems to be more sensitive to interferences than LC-220 and should only be considered for less complex samples.

In addition, we tested two commonly used colorimetric assays for protein determination, the BCA and the Bradford assay. Most of these assays are sold as commercial kits and often applied due to speed and ease of implementation. Figure 1 shows the results of high-purity BSA used as sample and calibrator. Under these controlled conditions and the calibration with the same protein, the tested colorimetric assays exhibited accuracy and precision similar to amino acid analysis. Some LODs of these methods are summarized in Table 1.

In the case of an unknown matrix and sample composition, however, common methods for the determination of the protein content can lead to severe over- or underestimation. As an example, the popular method based on the UV absorbance of proteins may not be applicable due to strong interferences of other UV-absorbing compounds. Separation on a reversed-phase column leads to the removal of many interfering compounds and delivered quite useful results. This method, however, needs some application-dependent validation. As a robust and widely applicable alternative, we suggest the use of aromatic amino acid analysis based on phenylalanine, AAAA(Phe), particularly for proteins with known sequence, AA composition or Phe content. AAAA based on tyrosine is even more sensitive and an excellent choice provided that the tyrosine content is not affected by severe chemical modifications such as oxidation or nitration, which is rarely the case. These methods are now sucessfully used in the authors’ labs for different purposes. The protein content is determined by application of the respective amino-acid-to-protein conversion factors, similar to the Jones factor used in food chemistry to convert nitrogen content (e.g., by Kjeldahl) into protein values. If the amino acid composition of the sample is unknown, the conversion factor of BSA or other pure proteins can be used as default values. Under such conditions, however, it should be clearly stated that BSA equivalents (or relative values thereof) have been determined. For even higher demands of accuracy, traditional amino acid analysis (AAA) could be performed for some representative samples. The experimentally determined Tyr and Phe content can be used to calculate specific amino-acid-to-protein conversion factors, which can be used in AAAA. The use of colorimetric methods for environmental samples is not recommended due to high overestimation, in our cases up to a factor of ten. Finally, a potential pitfall of the amino acid-based methods should be mentioned: Obviously, they comprise not only proteins, but free amino acids and small peptides, too. If this is undesirable, nanofiltration, dialysis or size exclusion chromatography (SEC) needs to be performed as a sample preparation step.

## Electronic supplementary material

Below is the link to the electronic supplementary material.

(PDF 2.33 MB)
