# Supplementary material for: Determination of the protein content of complex samples by aromatic amino acid analysis, liquid chromatography-UV absorbance, and colorimetry
Source: Anal Bioanal Chem. 2022 Mar 23;414(15):4457–70. doi: 10.1007/s00216-022-03910-1 (PMC9142416; doi:10.1007/s00216-022-03910-1)
Supplement: Supplementary file 1 — (PDF 2.33 MB) [file 216_2022_3910_MOESM1_ESM.pdf]

Analytical and Bioanalytical Chemistry
Electronic Supplementary Material

Determination of the protein content of complex samples by aromatic amino acid analysis, liquid chromatography-UV absorbance, and colorimetry

Kathrin Reinmuth-Selzle[1], Teodor Tchipilov[2], Anna T. Backes[1], Georg Tscheuschner[2], Kai Tang[1], Kira Ziegler[1], Kurt Lucas[1], Ulrich Pöschl[1], Janine Fröhlich-Nowoisky[1] and Michael G. Weller[2,*]

[1]Multiphase Chemistry Department, Max Planck Institute for Chemistry, 55128 Mainz, Germany

[2]Division 1.5 Protein Analysis, Federal Institute for Materials Research and Testing (BAM), 12489 Berlin, Germany

*correspondence to michael.weller@bam.de

1 Protein characterization

To characterize the six test proteins, SDS-PAGE and MALDI-TOF MS measurements were performed (Figure S6, S7).

A high-purity, low endotoxin, low IgG, monomeric bovine serum albumin (BSA) crystallized powder preparation with near native albumin characteristics was used. An aqueous solution of this BSA is termed high-purity BSA thereafter and was used as a secondary reference (1.07 mg/mL determined by AAAA(Phe)). The expected mass is 66.4 kDa, which was confirmed by MALDI-TOF MS measurements, and also the SDS-PAGE showed a single protein band at $\sim$65 kDa. The integrated LC-220 chromatogram gave a purity result of 98.8 % (related to the UV signal). Of note is that the LC-220 protein purity does not account for any inorganic or other non-absorbing material.

Chicken avidin forms a strong non-covalent complex with biotin, and is often used in biochemical assays such as ELISA or western blot [1]. Avidin is a basic protein with an isoelectric point of 10.0-10.5, and approx. 10 % of its total mass results from carbohydrates. The expected mass from the protein sequence is 13.9 kDa, and MALDI-TOF MS gave a mass of 15.7 kDa. SDS-PAGE showed one protein band at $\sim$17 kDa. The integrated LC-220 chromatogram gave a purity result of 87 %.

Myoglobin is an essential hemoprotein in striated muscle [2]. Myoglobin is a single chain heme protein with no disulfide bridges or free SH-groups and an iron content of 0.25-0.32 % (information of the manufacturer). The expected mass is 16.9 kDa, which was confirmed by MALDI-TOF MS measurements; the SDS-PAGE showed one protein band at $\sim$16 kDa. The integrated LC-220 chromatogram gave a purity result of 96.6 %.

Jacalin, a galactose-binding lectin from jackfruit seeds is able to bind to O-linked glycoproteins, particularly human IgA, which makes it useful for isolating plasma glycoproteins, investigating IgA nephropathies, and tumor detection [3]. The expected mass is 16.2 kDa, which was confirmed by MALDI-TOF MS. The SDS-PAGE showed two protein bands between $\sim$15-17 kDa. The integrated LC-220 chromatogram gave a purity result of 82.3 %.

Transferrins are iron-transport proteins [4]. The iron-deficient transferrin is called apotransferrin. We used bovine apotransferrin (Apo) in form of a sterile filtered lyophilized sample with a purity of 95 % and an iron content $<$ 40 ppm (information of the manufacturer). The expected mass is 75.8 kDa, and the measured MALDI-TOF MS gave a mass of 77.6 kDa. The SDS-PAGE showed two protein bands between $\sim$50-80 kDa. The integrated LC-220 chromatograms gave a purity result of 96.5 %.

Protein G is a single non-glycosylated protein, which can bind to a broad range of mouse and human IgG subclasses. In our study, the recombinant form of Protein G is used [5]. The expected mass of recombinant protein G (rPG) is 21.8 kDa, which was confirmed by MALDI-TOF MS measurements, and the SDS-PAGE showed several protein bands between $\sim$28-35 kDa. The integrated LC-220 chromatogram gave a purity result of 99.1 %.

According to our quality evaluation of the investigated proteins (SDS-PAGE, MALDI-TOF MS, LC-220), aliquots of the same aqueous high-purity BSA solution were used as our calibration solution with a concentration of 1.07 ± 0.03 mg/mL determined by AAAA(Phe). If not stated differently, all method calibrations were done with this high-purity BSA solution.

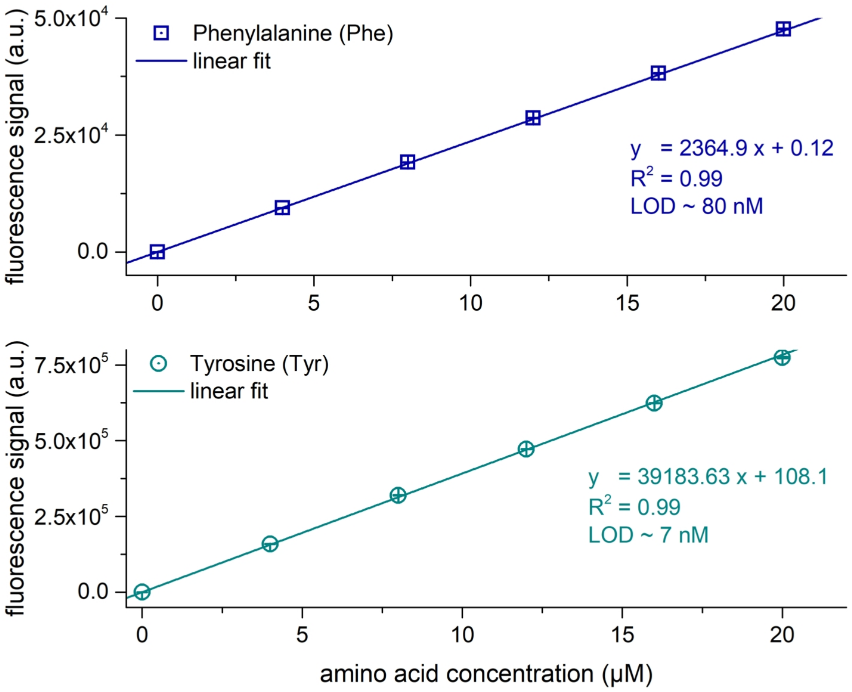

Fig. S1 Phenylalanine and tyrosine calibration lines for AAAA(Phe) and AAAA(Tyr). Fluorescence of Tyr and Phe were detected at 272 nm excitation/303 nm emission and 260 nm excitation/280 nm emission wavelengths, respectively.

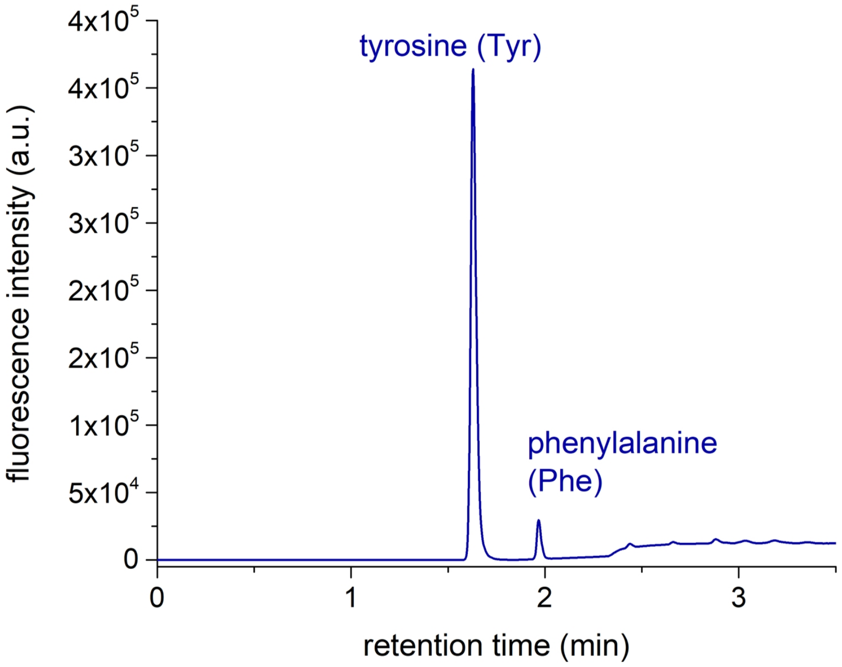

Fig. S2 UHPLC-FLD measurement for AAAA(Phe) and AAAA(Tyr). Phenylalanine (Phe) and tyrosine (Tyr) standards had a concentration of 20 µM. Fluorescence of Tyr and Phe was detected at 272 nm excitation/303 nm emission and 260 nm excitation/280 nm emission wavelengths, respectively.

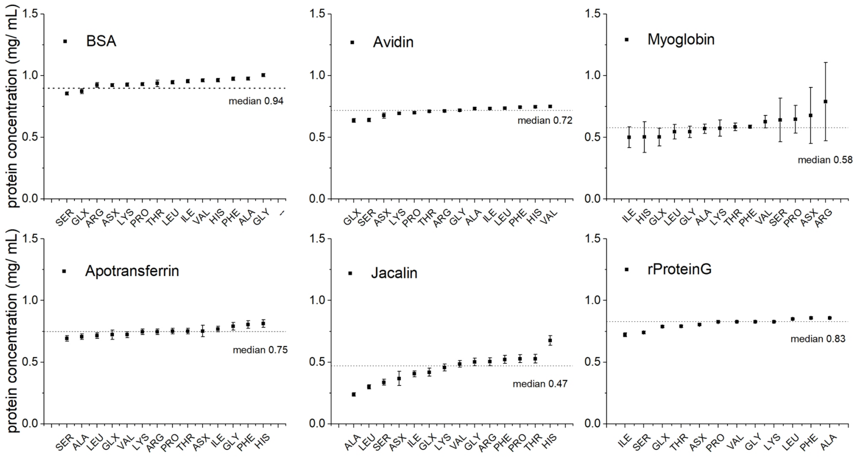

Fig. S3 Protein concentrations determined by AAA calculated for each amino acid

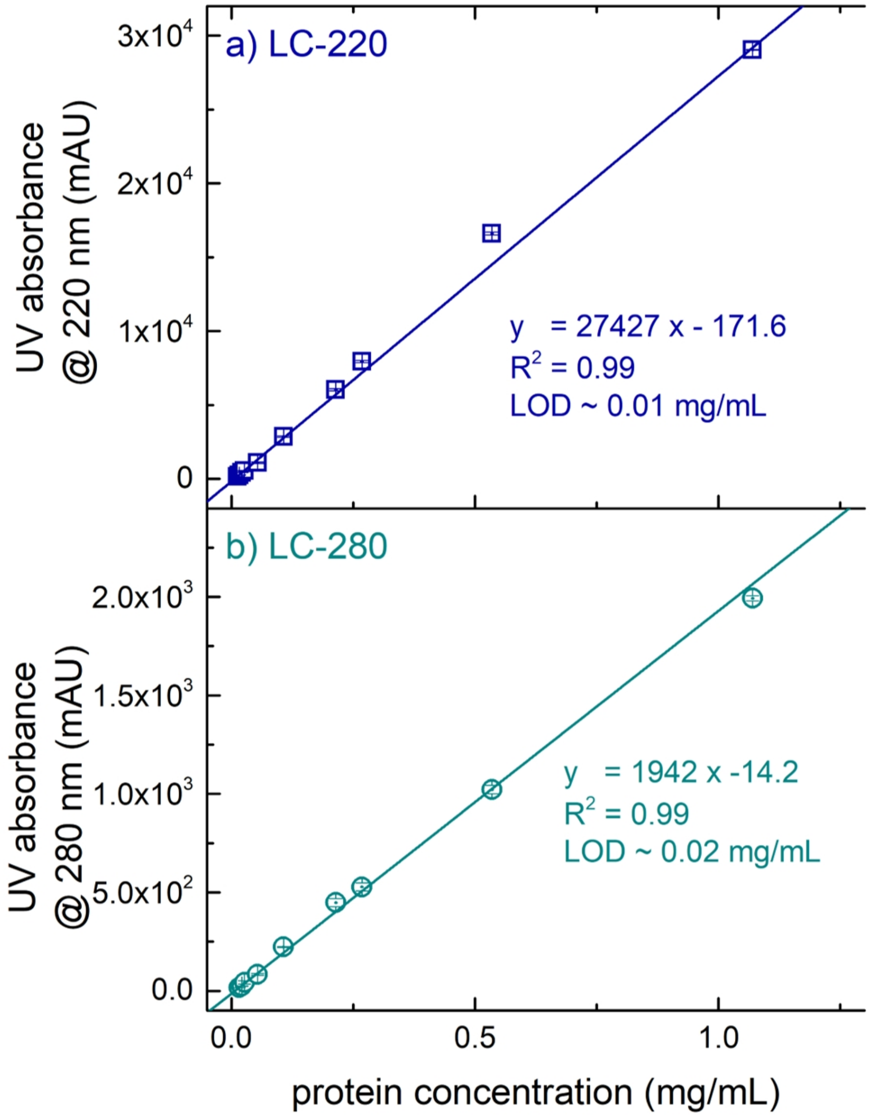

Fig. S4 Bovine serum albumin (BSA) calibration lines for LC-220 (a) and LC-280 (b)

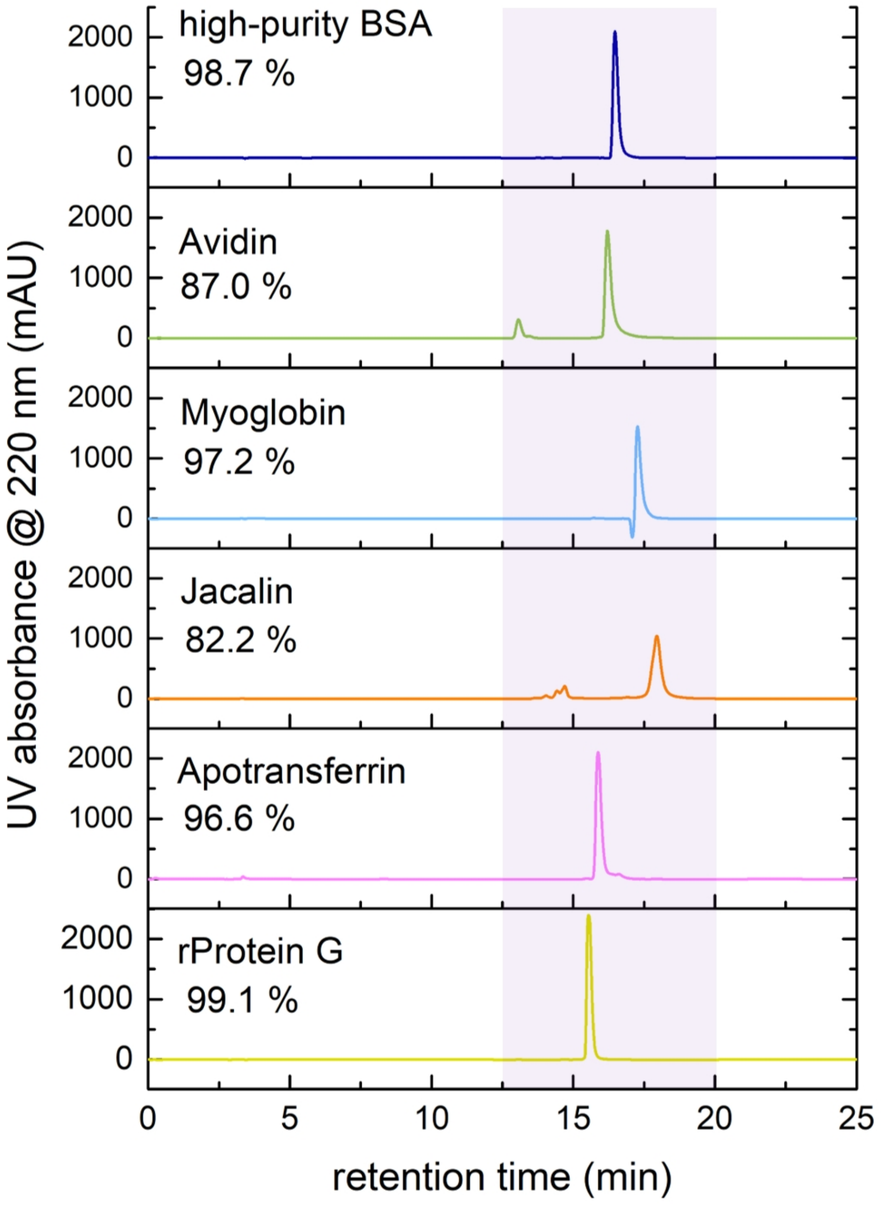

Fig. S5 Reversed-phase chromatograms at 220 nm (LC-220) of the tested proteins. The main peak area of the integrated chromatograms was used for the purity calculation. All protein peaks had retention times between 12.5 and 20 min (shaded area).

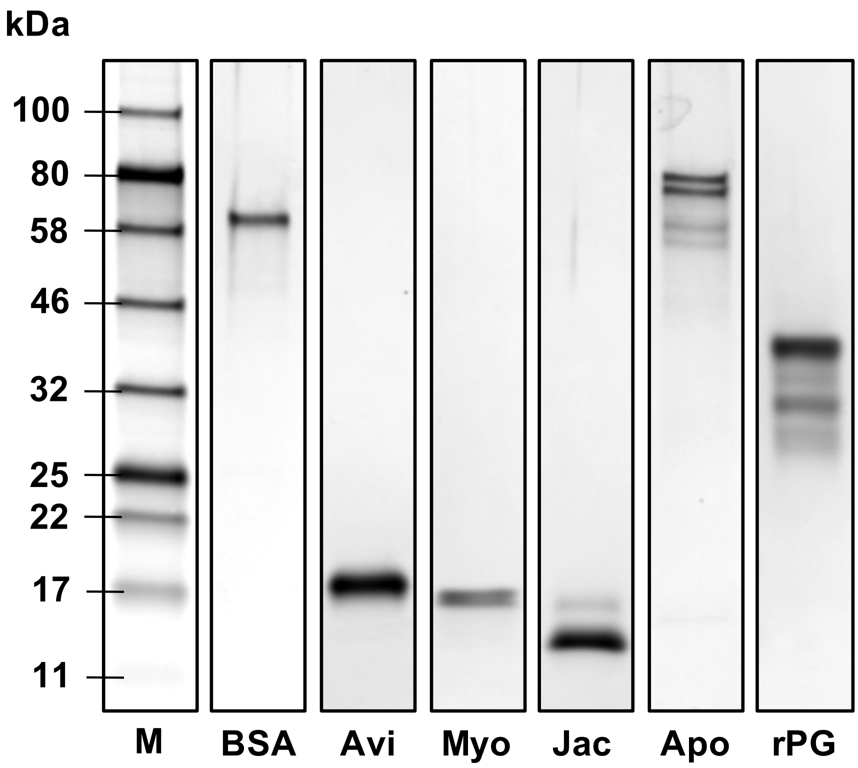

Fig. S6 SDS-PAGE gel of the six test proteins. Marker (M), bovine serum albumin (BSA), avidin (Avi), myoglobin (Myo), jacalin (Jac), apotransferrin (Apo), recombinant protein G (rPG)

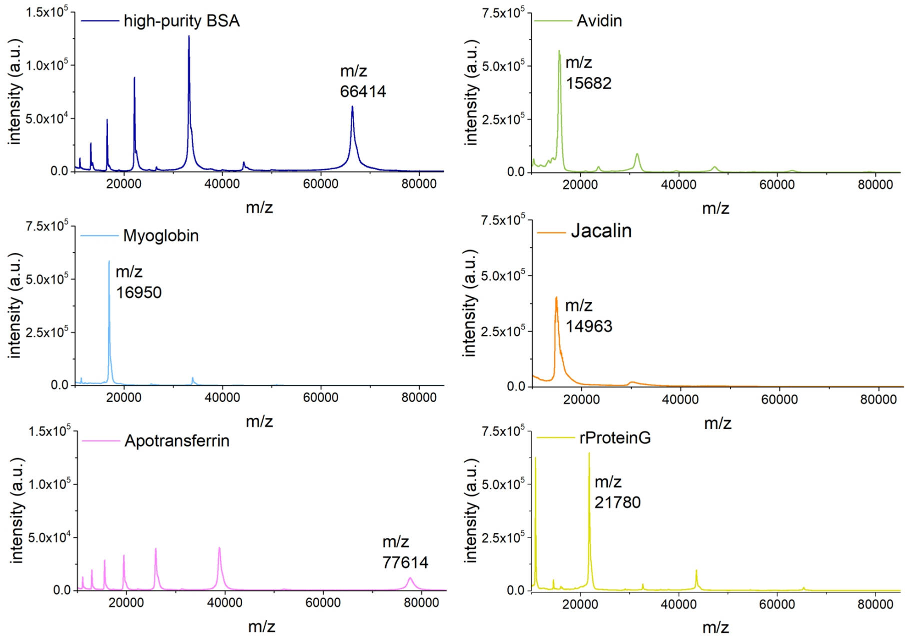

Fig. S7 MALDI-TOF-MS spectra of six test proteins: high-purity bovine serum albumin (BSA), avidin, myoglobin, jacalin apotransferrin, recombinant protein G.

References

1. Bratthauer GL. In: The Avidin–Biotin Complex (ABC) Method and other Avidin–Biotin Binding Methods. Totowa, NJ: Humana Press; 2010. p. 257–270.
2. Ordway GA, Garry DJ. Myoglobin: an essential hemoprotein in striated muscle. J Exp Biol. 2004;207(20):3441–3446.
3. Nascimento L, Silva DL, Pereira TB, Goncalves GRF, Verissimo LAA, Veloso CM, et al. Capture of lectins from jackfruit (Artocarpus integrifolia) seeds in a single step using a supermacroporous ion exchange cryogel. Rev Mex Ing Quim. 2019;18(1):313–324.
4. Crichton RR, Charloteaux-Wauters M. Iron transport and storage. Eur J Biochem. 1987;164(3):485–506.
5. Choe W, Durgannavar TA, Chung SJ. Fc-Binding Ligands of Immunoglobulin G: An Overview of High Affinity Proteins and Peptides. Materials. 2016;9(12):17.
